# Isolated, Unilateral Inguinal Tuberculous Lymphadenitis

**DOI:** 10.4269/ajtmh.18-0211

**Published:** 2019-04

**Authors:** Isabel Ramírez

**Affiliations:** 1 Internal Medicine, Infectious Diseases, Hospital Pablo Tobón Uribe, Medellín, Colombia;; 2 Universidad de Antioquia, Medellín, Colombia

**Figure 1.**
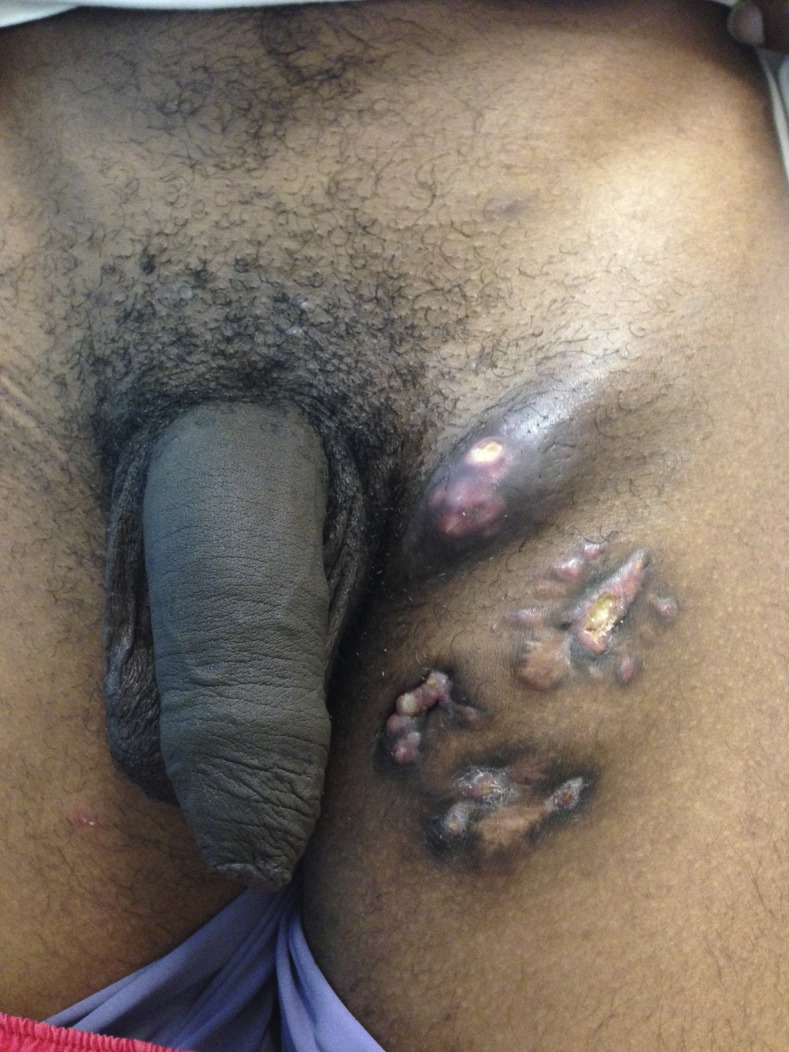
Isolated unilateral tuberculous lymphadenitis. This figure appears in color at www.ajtmh.org.

**Figure 2.**
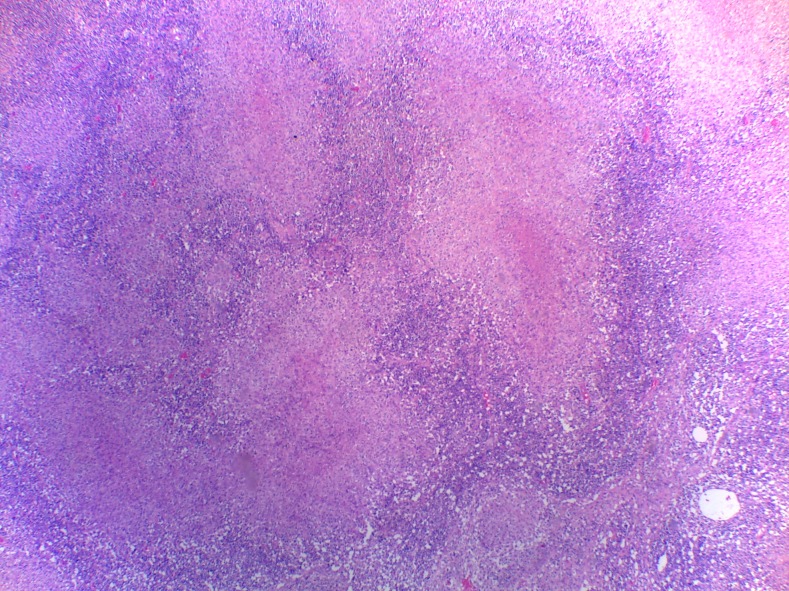
Histologic examination of lymph node tissue. Hematoxylin and eosine stain showing multiple granulomas with multinucleated giant cell (×100). This figure appears in color at www.ajtmh.org.

**Figure 3.**
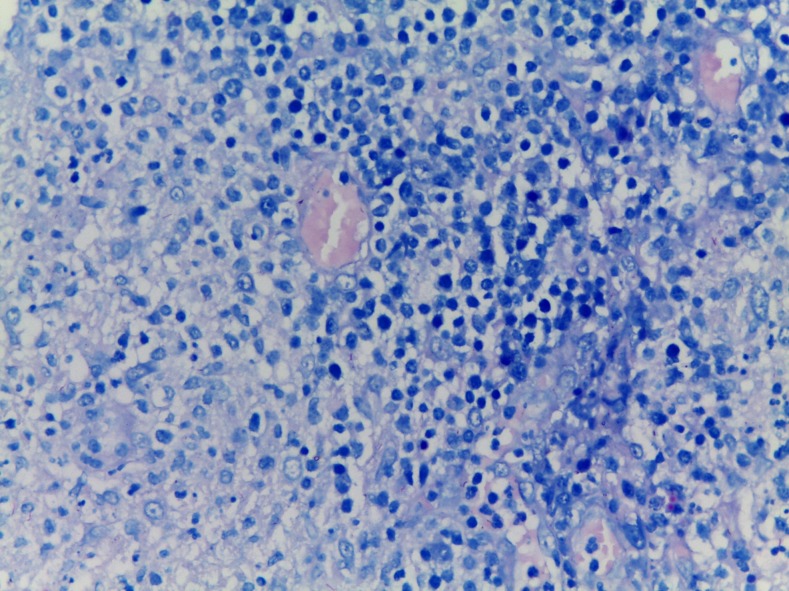
Ziehl-Neelsen stain showing asid-fast bacilli of *Mycobacterium tuberculosis* (×100). This figure appears in color at www.ajtmh.org.

A 17-year-old heterosexual man presented with a 3-month history of a painless, enlarging inguinal lymph node and multiple discharging sinuses. There had been no clinical improvement despite multiple empirical antibiotic treatments for other infections which share similar clinical presentations, such as lymphogranuloma venereum. Specific details in reference to diagnosis and treatment could not be obtained (Figure 1). There was no history of recent unprotected sexual intercourse, previous tuberculosis (TB) exposure, symptoms suggestive of other systemic illnesses, fever, weight loss, cough, sore throat, urethral discharge, genital ulcer, or trauma to the lower extremities. On physical examination, an enlarged, 4 × 3-cm inguinal lymph node was noted; the remainder of the examination was unremarkable. The chest X-ray showed no abnormalities and a test for human immunodeficiency virus was negative. The patient was evaluated for syphilis, but not for other sexually transmitted diseases, given that he had no history of recent unprotected sexual exposure, no urethral discharge, or genital ulcer. Histologic examination of tissue obtained by an excisional biopsy showed granulomas with multinucleated giant cells (Figure 2) and acid-fast bacilli (Figure 3); culture grew *Mycobacterium tuberculosis*. Standard antituberculous treatment for 6 months was initiated. Clinical response was observed after 2 months of treatment.

In the initial diagnosis of inguinal lymphadenitis, it is important to consider the potential etiologies, both infectious and noninfectious diseases. Painless lymphadenitis is usually due to toxoplasmosis, cat-scratch disease, syphilis, and TB; however, it can be painful at the beginning, and it can occur bilaterally. Painful unilateral lymphadenopathy can be caused by *Chlamydia trachomatis* and *Haemophilus ducreyi*; however, these are usually associated with urethral discharge or a genital ulcer. Other potential etiologies include secondary bacterial infection of genital scabies or pediculosis pubis, plague, pyogenic infection of the leg, atypical mycobacterial infections, and persistent generalized lymphadenopathy of AIDS. Noninfectious causes must also be considered, such as metastatic lymph node lesions and lymphoma.

The initial evaluation should include a history of recent sexual contact and testing for syphilis, *Cytomegalovirus*, Epstein–Barr virus, *Toxoplasma gondii*, hepatitis B and C virus, herpes simplex virus, and HIV. Cervical cultures for *Neisseria gonorrhoeae* and *Ureaplasma urealyticum*, polymerase chain reaction (PCR) for *Mycoplasma hominis*, and nucleic acid amplification test for *C. trachomatis* should be obtained if the patient is sexually active. Empirical treatment is not recommended given that the causes can be multiple; therefore, it is always important to make the etiological diagnosis.

Tuberculous lymphadenitis is the most common extrapulmonary manifestation of TB; it comprises 30–50% of these cases.^1^ Cervical lymph nodes are involved in 57% of cases, supraclavicular lymph nodes in 26%, submandibular lymph nodes in 3%, and axillary lymph nodes in 12%.^2,3^ Up to 17% of cases are bilateral and 78% of cases involve between one and three nodes. Isolated inguinal tuberculous lymphadenitis is infrequent, representing up to 8% of cases in all reported series.^2,4^ Active pulmonary TB occurs infrequently in immunocompetent patients with TB lymphadenitis; however, up to 15% of tuberculous lymphadenitis is associated with pulmonary TB.^5^ Therefore, a chest X-ray must be performed as part of initial evaluation, and additional tests must be carried out based on clinical symptoms and findings. A fine needle aspiration is essential to make the diagnosis in tuberculous lymphadenopathy, as it can reveal granulomas in 61% of cases and a positive culture in 62%; for surgical specimens, the diagnostic yield is 88% and 71%, respectively.^3,4^ A 6-month TB treatment regimen is recommended as standard therapy. In countries with a high prevalence of mycobacterial resistance, a four-drug treatment regimen (isoniazid, rifampicin, pyrazinamide, and ethambutol) should be preferred within the first 2 months.
